# Prescription Trends in the Management of Lower Urinary Tract Symptoms in Men: An Online Survey of Indian Urologists

**DOI:** 10.7759/cureus.115189

**Published:** 2026-08-25

**Authors:** Prakash Patwardhan, Santosh Palkar, Rahul Sharma, Sanjay Kamble, Shubhra Dhurandhar

**Affiliations:** 1 Urology, Sapna Clinic, Pune, IND; 2 Urology, Zen Multi Speciality Hospital, Mumbai, IND; 3 Urology, ILS Hospital, Agartala, IND; 4 Medical Affairs, Samarth Life Sciences Pvt Ltd, Mumbai, IND

**Keywords:** benign prostatic hyperplasia, fixed drug combination, lower urinary tract symptoms, muscarinic antagonists, practice patterns, α1-blockers

## Abstract

Background

Lower urinary tract symptoms (LUTS), highly prevalent among aging men, adversely affect the quality of life. Pharmacological management has evolved with multiple drug classes, but real-world prescribing patterns in India have not been adequately studied. Thus, we evaluated prescription trends among Indian urologists for the management of male LUTS, assessed symptom patterns, evaluated factors influencing choice of fixed-dose combination (FDC), with special focus on silodosin-mirabegron FDC, and identified adverse events (AEs) and adherence.

Methods

This cross-sectional, questionnaire-based survey was performed over three months (January 2, 2026, to April 5, 2026) and included 110 Indian urologists actively managing male patients with LUTS. A 27-item structured questionnaire captured demographic characteristics, clinical practice patterns, and prescribing preferences for LUTS pharmacotherapy, including monotherapies, combination therapies, and FDCs, along with AEs and adherence.

Results

Of 135 urologists invited, 110 (81.48%) completed the survey. LUTS mainly involved a combination of storage, voiding, and post-micturition symptoms (n = 59, 53.6%). As a class, α1-blockers were the most frequently prescribed monotherapy (n = 109, 99.1% frequent/very frequent use), with silodosin (n = 98, 89.1%) and tamsulosin (n = 84, 76.4%) being most preferred. Use of dutasteride (n = 108, 98.2%) exceeded finasteride (n = 13, 11.8%). In patients with benign prostatic hyperplasia (BPH) with overactive bladder, silodosin-mirabegron was the most favored FDC (n = 97, 88.2%). FDCs of α1-blockers with muscarinic receptor antagonists and β-3 adrenergic agonists were preferred due to their proven efficacies (n = 83, 75.5% and n = 87, 79.1%, respectively) and lower incidences of AEs (n = 69, 62.7% and n = 80, 72.7%). Common AEs included dry mouth (n = 73, 66.4%), anejaculation (n = 69, 62.7%), and headache (n = 67, 60.9%). Moderate adherence (25%-75%) was reported by 95 (86.4%) urologists.

Conclusion

Adherence to pharmacotherapy, as assessed by participating urologists, was moderate, with urologists preferring silodosin as first-line therapy and silodosin-mirabegron FDC for mixed LUTS.

## Introduction

Lower urinary tract symptoms (LUTS) include a range of symptoms, including storage, voiding, and post-micturition, which negatively affect quality of life (QoL) in aging men [1]. Globally, benign prostatic hyperplasia (BPH) remains the primary etiologic factor, with an estimated pooled prevalence of 26.2% [2], while overactive bladder (OAB) affects around 11.8% of men, increasing considerably with age [3]. India shares a significant burden, with community-based studies reporting LUTS suggestive of BPH in 10.3% of men aged 50 years or more, with age-specific prevalence increasing from 25% to over 50% in men aged 40-49 years to 70-79 years, respectively [4,5]. The most bothersome LUTS include nocturia, frequency, and urgency that interfere with routine activities, work productivity, and sleep [6].

Current guidelines from professional bodies, including the European Association of Urology (EAU) [7], American Urological Association (AUA) [8], Urological Society of India [9], and French LUTS Committee [10], recommend α1-blockers as first-line agents for moderate-to-severe LUTS, while 5α-reductase inhibitors (5-ARIs) are added when prostate volume exceeds 40 mL. Combination therapy involving α1-blockers and 5-ARIs reduces the risk of acute urinary retention by 81% compared to placebo, while 35% and 68% risk reduction were observed with doxazosin and finasteride, respectively [11]. Antimuscarinics or β-3 agonists (e.g., mirabegron) are recommended for predominant storage symptoms, while tadalafil is added in the presence of comorbid erectile dysfunction [7,10].

Despite the availability of various guidelines and their frequent updates, real-world prescription patterns vary substantially. Literature suggests low adherence to BPH pharmacotherapy, with one-year adherence rates ranging from 35% for α1-blockers to 9% for combination therapy with 5-ARIs [12]. Compared to separate tablets, use of fixed-dose combinations (FDCs) is reported to have significantly greater and longer duration of persistence [13]. However, such data remain scarce from low- and middle-income countries. A recent survey from India reported that silodosin is widely prescribed for BPH, especially in those with comorbidities, while mirabegron is used as a first-line therapy for managing OAB [14]. In spite of that, systematic evidence on prescribing trends, factors influencing selection of FDC, and adherence challenges in the context of Indian settings is lacking. Thus, the primary objective of the survey was to evaluate prescription trends among Indian urologists for the management of male LUTS. Meanwhile, the secondary objective was to evaluate symptom patterns and factors influencing choice of FDC and identify adverse events (AEs) as well as adherence challenges reported by participating urologists.

## Materials and methods

Study design and ethics

This cross-sectional multiple-response online questionnaire-based survey was performed over a period of three months (January 2, 2026, to April 5, 2026). The study was approved by the Independent Ethics Committee (CIEC/2025/0408; dated December 29, 2025), and informed consent was obtained from urologists after explaining the purpose and benefits of the study.

Study population

The survey included 110 urologists across India who actively managed male patients with LUTS/BPH in their clinic, had at least two years of independent clinical practice post-residency/fellowship, and routinely managed and prescribed medication for a minimum of five new or existing male LUTS patients per month. Urologists were excluded if they treated fewer than five male LUTS/BPH patients per month, did not have prescribing authority for LUTS/BPH, and did not respond to the complete survey questionnaires.

Study procedure

A structured questionnaire was developed to evaluate the knowledge and practice of urologists associated with medical management of LUTS/BPH in men (Appendix). Questions were designed as multiple-choice to facilitate response standardization. The urologists prescribing medication for LUTS/BPH were recruited via convenience sampling from professional networks and their referrals and invited to take part in the survey. It was reviewed by two independent urologists for content and face validity, relevance, and clarity. However, as the questionnaire was designed for assessment of descriptive practice patterns without scoring or grading, it was not formally validated or pilot-tested. The urologists who accepted the invitation were briefed about the study details and asked to sign an informed consent form. After obtaining consent, the urologists were asked to complete an online questionnaire-based survey. This online questionnaire-based survey was conducted using Google Forms (Google, Mountain View, CA, USA), and only fully completed responses were included in the analysis. Upon completion of the survey, the response rate was calculated, and data were analyzed.

The survey collected demographic and professional information of urologists, including their sex, location of practice, and years of clinical practice. Moreover, information regarding presentation of patients with LUTS and their management, including frequency of managing men with LUTS, symptoms and etiologies of LUTS, pharmacological options preferred for managing these patients, drug(s) preferred within each available class, FDCs preferred in patients presenting with BPH and associated OAB, reasons for preferring FDC of muscarinic receptor antagonists (MRAs) and β-3 adrenergic agonists (mirabegron) for LUTS, and AEs, adherence, and feedback of patients regarding the tolerability of silodosin-mirabegron FDC, was collected.

Survey response rate

The survey response rate was determined by dividing the number of urologists who responded by the total number of urologists who participated. An 80% or greater response rate was taken into consideration for this survey [15]. Telephonic and in-person informed consent was obtained, and a questionnaire was sent to 135 urologists, of which 110 completed the survey, resulting in a response rate of 81.48%.

Statistical analyses

Due to the use of descriptive statistics, no inferential analyses were planned. Continuous variables were represented as mean values ± standard deviation (SD) or median values with range, while categorical variables were represented as percentages. Microsoft 365 Excel (Redmond, WA, USA) was used for performing descriptive analysis.

## Results

All surveyed urologists were male (n = 110, 100%) and mainly practiced in non-metropolitan regions (n = 95, 86.4%). Most of the urologists had 5-10 years (n = 49, 44.5%) and 10-15 years (n = 34, 30.9%) of experience (Table 1).

**Table 1:** Demographic characteristics Data represented as frequency (percentage)

Characteristics	n = 110	%
Sex, n (%)		
Male	110	100
City of practice, n (%)		
Metropolitan	15	13.6
Non-metropolitan	95	86.4
Years of clinical practice, n (%)		
<5 years	9	8.2
5-10 years	49	44.5
10-15 years	34	30.9
15-20 years	9	8.2
>20 years	9	8.2

The majority of urologists reported that LUTS are very common in clinical practice (n = 76, 69.1%). The most frequently reported LUTS was a combination of storage, voiding, and post-micturition symptoms (n = 59, 53.6%), followed by mixed storage and voiding symptoms (n = 26, 23.6%). BPH with OAB (n = 56, 50.9%) and BPH alone (n = 25, 22.7%) were the most common etiologies of LUTS (Table 2).

**Table 2:** Clinical perspectives on prevalence, symptom patterns, and etiologies Data represented as frequency (percentage) LUTS: lower urinary tract symptoms; BPH: benign prostatic hyperplasia; OAB: overactive bladder

Characteristics	n = 110	%
How common is LUTS in men in clinical practice?		
Not common	1	0.9
Common	33	30.0
Very common	76	69.1
Which LUTS are most frequently reported by men in clinical practice?		
Storage symptoms	11	10.0
Voiding symptoms	13	11.8
Storage plus voiding symptoms	26	23.6
Voiding plus post-micturition symptoms	1	0.9
All of the above	59	53.7
What are the commonly attributed etiologies of LUTS in men observed in clinical practice?		
BPH	25	22.7
BPH with OAB	56	50.9
BPH with bladder infections	3	2.7
BPH with OAB and bladder infections	8	7.3
BPH with OAB, bladder infections, and bladder pain syndrome	5	4.5
Bladder pain syndrome	1	0.9
OAB	6	5.6
OAB with bladder infections and bladder pain syndrome	3	2.7
Bladder infections and bladder pain syndrome	3	2.7

Of various pharmacological classes surveyed, α1-blockers were the most frequently prescribed monotherapy for men with LUTS, with nearly all urologists reporting very frequent (n = 77, 70.0%) or frequent (n = 32, 29.1%) use. Among other classes, 5-ARIs (n = 72, 65.5%), MRAs (n = 86, 78.2%), β-3 adrenergic agonists (n = 71, 64.5%), and phosphodiesterase 5 inhibitors (PDE5Is, n = 75, 68.2%) were frequently used, while selective serotonin and norepinephrine reuptake inhibitors (SNRIs) were less frequently (n = 40, 36.4%) and rarely (n = 41, 37.3%) used. Combination therapies of α1-blockers with 5-ARIs (n = 73, 66.4%), MRAs (n = 79, 71.8%), β-3 adrenergic agonists (n = 65, 59.1%), and PDE5Is (n = 69, 62.7%) were frequently used. Though the combination of β-3 agonists with MRAs was frequently used (n = 66, 60.0%), its combination with SNRIs was less frequently used (n = 48, 43.6%; Table 3).

**Table 3:** Prescribing patterns of pharmacological therapies Data represented as frequency (percentage) 5-ARIs: 5α-reductase inhibitors; MRAs: muscarinic receptor antagonists; PDE5Is: phosphodiesterase 5 inhibitors; SNRIs: serotonin and norepinephrine reuptake inhibitors; LUTS: lower urinary tract symptoms

Drug class	Frequency of prescription (n = 110)							
	Very frequent		Frequent		Less frequent		Rare	
	n	%	n	%	n	%	n	%
How frequently are various pharmacological options preferred for men presenting with LUTS?								
α1-blockers	77	70.0	32	29.1	0	0.0	1	0.9
5-ARIs	28	25.4	72	65.5	10	9.1	0	0.0
PDE5Is	10	9.1	75	68.2	23	20.9	2	1.8
MRAs	14	12.7	86	78.2	10	9.1	0	0.0
β-3 adrenergic agonists	16	14.5	71	64.5	19	17.4	4	3.6
SNRIs	7	6.3	22	20.0	40	36.4	41	37.3
α1-blockers-5-ARIs	25	22.7	73	66.4	9	8.2	3	2.7
α1-blockers-MRAs	12	10.9	79	71.8	18	16.4	1	0.9
α1-blockers-β-3 adrenergic agonist	18	16.4	65	59.1	22	20.0	5	4.5
α1-blockers-PDE5Is	17	15.5	69	62.7	20	18.2	4	3.6
β-3 agonist-MRAs	18	16.3	66	60.0	19	17.3	7	6.4
β-3 agonist-SNRIs	7	6.4	31	28.2	48	43.6	24	21.8

Within drug classes, silodosin (n = 98, 89.1%) and tamsulosin (n = 84, 76.4%) were the most preferred α1-blockers, while, among 5-ARIs, dutasteride (n = 108, 98.2%) was overwhelmingly favored over finasteride (n = 13, 11.8%). Use of solifenacin (n = 103, 93.6%) dominated among MRAs, and mirabegron (n = 108, 98.2%) was the clear choice within β-3 adrenergic agonists. Tadalafil (n = 102, 92.7%) was strongly preferred over sildenafil (n = 11, 10.0%) among PDE5Is, and duloxetine (n = 107, 97.3%) was the predominantly used SNRI. Among FDCs for BPH with associated OAB, silodosin-mirabegron (n = 97, 88.2%) was the most favored (Table 4).

**Table 4:** Preferred drugs and fixed-dose combinations for management Data represented as frequency (percentage) 5-ARIs: 5α-reductase inhibitors; MRAs: muscarinic receptor antagonists; PDE5Is: phosphodiesterase 5 inhibitors; SNRIs: serotonin and norepinephrine reuptake inhibitors; LUTS: lower urinary tract symptoms; BPH: benign prostatic hyperplasia; OAB: overactive bladder

Drug class	n = 110	%
Which drug(s) are preferred within each class for men presenting with LUTS in clinical practice?		
α1-blockers		
Tamsulosin	84	76.4
Silodosin	98	89.1
Alfuzosin	46	41.8
Terazosin	1	0.9
5-ARIs		
Dutasteride	108	98.2
Finasteride	13	11.8
MRAs		
Solifenacin	103	93.6
Fesoterodine	23	20.9
Darifenacin	17	15.5
Tolterodine	44	40.0
Oxybutynin	14	12.7
Trospium	5	4.5
Mirabegron	1	0.9
β-3 adrenergic agonists		
Mirabegron	108	98.2
Vibegron	5	4.5
PDE5Is		
Sildenafil	11	10.0
Tadalafil	102	92.7
SNRIs		
Duloxetine	107	97.3
Venlafaxine	10	9.1
Dapoxetine	1	0.9
Which of the available fixed-dose combinations are preferred for patients presenting with BPH and associated OAB?		
Silodosin-mirabegron	97	88.2
Silodosin-dutasteride	15	13.6
Alfuzosin-tadalafil	17	15.5
Alfuzosin-dutasteride	8	7.3
Alfuzosin-solifenacin	1	0.9
Tamsulosin-dutasteride	20	18.2
Tamsulosin-tadalafil	20	18.2
Tamsulosin-finasteride	2	1.8
Solifenacin-tamsulosin	1	0.9
Silodosin-dutasteride-mirabegron-solifenacin	1	0.9

Proven efficacy was the main reason for preferring both MRAs (n = 83, 75.5%) and β-3 adrenergic agonists (n = 87, 79.1%) in FDC regimens. A lower incidence of adverse effects was also a key factor, reported by 69 (62.7%) for MRAs and 80 (72.7%) for beta-3 agonists. Safety in patients with narrow-angle glaucoma or cognitive impairment influenced choice in 50 (45.5%) and 60 (54.5%) urologists, respectively (Table 5).

**Table 5:** Reasons for preference of muscarinic receptor antagonists and β-3 adrenergic agonists in fixed-dose combination regimens Data represented as frequency (percentage)

Characteristics	n = 110	%
Muscarinic receptor antagonists		
Cost-effective	24	21.8
Proven efficacy	83	75.5
Widely available	28	25.5
Failure of monotherapy	44	40.0
Safer in patients with narrow-angle glaucoma or cognitive impairment	50	45.5
Lesser incidence of adverse effects, including constipation and dry mouth	69	62.7
β-3 adrenergic agonists (mirabegron)		
Cost-effective	22	20.0
Proven efficacy	87	79.1
Widely available	15	13.6
Failure of monotherapy	38	34.5
Safer in patients with narrow-angle glaucoma or cognitive impairment	60	54.5
Lesser incidence of adverse effects, including constipation and dry mouth	80	72.7

The most frequent AEs associated with the use of silodosin-mirabegron FDC were dry mouth (n = 73, 66.4%), anejaculation (n = 69, 62.7%), headache (n = 67, 60.9%), and dizziness (n = 52, 47.3%). Regarding adherence, 95 (86.4%) urologists reported that patients were moderately adherent to FDC (25%-75%). Despite AEs and moderate adherence, patient feedback was largely positive, with 51 (46.4%) urologists reporting a combination of improved QoL, reduced overall BPH-related symptoms, and good tolerability over an extended period, followed by a combination of improved QoL and reduced overall BPH-related symptoms (n = 37, 33.6%, Table 6).

**Table 6:** Tolerability, adherence, and patient-reported outcomes of combination therapy with silodosin and mirabegron Data represented as frequency (percentage) LUTS: lower urinary tract symptoms; BPH: benign prostatic hyperplasia

Characteristics	n = 110	%
Adverse events with combination therapy of silodosin and mirabegron		
Dry mouth	73	66.4
Anejaculation	69	62.7
Headache	67	60.9
Dizziness	52	47.3
Postural hypotension	43	39.1
Nasal stuffiness	40	36.4
Constipation	37	33.6
Hypertension	32	29.1
Palpitation	16	14.5
Obstructed flow	12	10.9
Adherence to the combination therapy of silodosin and mirabegron		
<25% patients	9	8.2
25%-50% patients	50	45.5
51%-75% patients	45	40.9
76%-100% patients	6	5.5
What is the feedback of patients with male LUTS regarding the tolerability of the combination therapy of silodosin and mirabegron compared to other combination therapies of muscarinic receptor antagonists?		
Improved quality of life	14	12.7
Reduction in overall BPH-related symptoms	6	5.5
Good tolerability over an extended period	1	0.9
Improved quality of life + reduction in overall BPH-related symptoms	37	33.6
Reduction in overall BPH-related symptoms + good tolerability over an extended period	1	0.9
All of the above	51	46.4

## Discussion

Among aging male populations, LUTS pose a significant clinical challenge, with considerable adverse impact on QoL [1]. Over a period of time, approval of various pharmacological classes, including α1-blockers, β-3 adrenergic agonists, 5-ARIs, MRAs, SNRIs, and PDE5Is, has led to substantial evolution in the management of male LUTS [8,10]. This survey provides an up-to-date understanding of real-world prescribing practices among Indian urologists, suggesting crucial trends that both align with and diverge from professional treatment guidelines.

The principal finding of this survey suggests that α1-blockers remain the foundation of LUTS pharmacotherapy, with 109 (99.1%) urologists confirming very frequent/frequent use. This is consistent with the EAU and the French LUTS Committee guidelines, which recommend α1-blockers as first-line therapy for men with moderate-to-severe LUTS [8,10]. The preference for silodosin (n = 98, 89.1%) over tamsulosin (n = 84, 76.4%) is a notable finding. This is consistent with the findings of an Indian expert survey, where 89.1% of specialists prescribe silodosin for BPH [16]. Being a highly selective α1A-blocker, silodosin is reported to have minimal vasodilatory properties, with a hypotensive effect identical to placebo [10]. Moreover, the selectivity of silodosin explains the lower incidence of cardiovascular AEs, though ejaculatory dysfunction remains a substantial consideration [10]. Meanwhile, a randomized controlled trial reported anejaculation rates of 73.9% with silodosin monotherapy, increasing to 84% when combined with mirabegron [17], attributed to differences in study population or pharmacodynamic interaction.

Among 5-ARIs, high preference for dutasteride (n = 108, 98.2%) over finasteride (n = 13, 11.8%) represents a significant shift from historical prescribing patterns. This finding is consistent with the available literature suggesting that dutasteride may lead to reduced risks of acute urinary retention and BPH-related surgery compared to finasteride [18]. Unlike finasteride, a selective type II 5-ARI, dutasteride inhibits both types I and II 5-ARIs and thus leads to nearly complete suppression of dihydrotestosterone (95%), likely describing its clinical preference despite comparable efficacy reported in the literature [10,19].

With reference to combination therapy of various classes, the frequent use of α1-blockers with MRAs (n = 79, 71.8%) and β-3 agonists (n = 65, 59.1%) suggests increasing awareness that storage symptoms usually dominate or persist even after adequate control of voiding symptoms [20]. A recent network meta-analysis concluded that combination therapies often show superior efficacy for urgency episodes and urge urinary incontinence than monotherapies [21]. The strong preference for mirabegron (n = 108, 98.2%) is consistent with the fact that mirabegron is widely used as a first-line treatment by Indian urologists [14]. Moreover, the efficacy of mirabegron is comparable to antimuscarinics without the risks of cognitive impairment, especially in elderly men [22].

High preference for silodosin-mirabegron FDC (n = 97, 88.2%) among surveyed urologists represents a unique finding. This is in agreement with the findings reported by another Indian survey that 82.5% of urologists preferred silodosin-mirabegron FDC [23]. This combination handles both components of mixed LUTS, with silodosin improving the voiding function by relaxing prostatic smooth muscle and mirabegron reducing urgency and frequency by increasing bladder storage capacity [7,10]. Moreover, preference for fixed-dose regimens of MRAs (n = 83, 75.5%) and β-3 agonists (n = 87, 79.1%) was primarily driven by their proven efficacy. The lower incidence of AEs was also crucial, mentioned by 69 (62.7%) and 80 (72.7%) urologists, respectively. This attention to tolerability is clinically significant, as low persistence still remains an important challenge in pharmacotherapy of LUTS [24,25]. The most frequently observed AEs with silodosin-mirabegron FDC, including dry mouth (n = 73, 66.4%), anejaculation (n = 69, 62.7%), headache (n = 67, 60.9%), and dizziness (n = 52, 47.3%), are consistent with known tolerability profiles of individual agents [10,26]. The high rate of anejaculation indicates uroselectivity of silodosin, as the incidence of ejaculatory dysfunction is significantly greater with silodosin than placebo [10].

Notwithstanding moderate adherence reported by 95 (86.4%) urologists (25%-75% adherence range), patient feedback mainly remained positive, with 51 (46.4%) reporting combined improvements in QoL and symptomatic improvement. This demonstrates that even partial adherence may result in clinically significant benefits. The moderate adherence rates, however, emphasize a continuing challenge; in real-world scenarios, persistence with BPH medications is 29% at one year, with even lower rates for combination therapy (9%) [11,25]. These findings suggest that efficacy by itself does not ensure the best possible compliance.

The most frequently observed LUTS pattern was a combination of storage, voiding, and post-micturition symptoms (n = 59, 53.6%), followed by a combination of storage and voiding symptoms (n = 26, 23.6%). Pure storage (n = 11, 10.0%) or voiding symptoms (n = 13, 11.8%) were seldom observed. This distribution highlights that multiple pathophysiologic mechanisms are often involved in LUTS in Indian men, which supports the justification for combination therapy that targets detrusor overactivity as well as bladder outlet obstruction [20].

However, certain findings deviate from prescribing practices observed in the Western world. Given that tadalafil for BPH is not always reimbursed in Indian healthcare settings, the comparatively lower preference for PDE5Is (n = 75, 68.2% frequent use) in comparison to European settings may be due to financial concerns [27]. Moreover, the restricted evidence base for LUTS indications is consistent with the limited use of SNRIs (n = 40, 36.4% less frequent, n = 41, 37.3% rarely used) [24].

Limitations

Due to the cross-sectional, questionnaire-based design, this survey has certain limitations. First, recall bias could have affected the responses, because urologists might underreport less preferred prescribing practices or overreport adherence to guidelines. Second, the sample size was small and mainly represented non-metropolitan practitioners (90%) with 5-15 years of experience, which may limit their applicability to more senior urologists or those practicing in metropolitan cities. Third, objective clinical parameters, including urodynamic results, prostate volume, and prostate-specific antigen levels, which have an impact on treatment choices, were not included in the survey. Fourth, the study relies entirely on physicians’ perceptions regarding AEs, adherence, and patient satisfaction, rather than objective patient-reported or clinical data. Fifth, the questionnaire was not formally validated or pilot-tested. Sixth, urologists were recruited through convenience sampling from professional networks and subsequent referrals, which may limit generalizability and introduce selection bias. Finally, the lack of assessment of patient-level outcomes, including actual adherence, persistence, and clinical response, represents a crucial area for further evaluation.

## Conclusions

This nationwide survey shows that Indian urologists prefer α1-blockers, especially silodosin, as the first-line monotherapy for LUTS in men. Among the urologists surveyed, strong preference for the silodosin-mirabegron FDC indicates increasing clinical confidence in dual-mechanism strategies that treat storage and voiding symptoms. In clinical practice, dutasteride has largely supplanted finasteride. Patient-reported outcomes are generally favorable, but adherence is still moderate. These findings provide opportunities for focused educational interventions to improve LUTS management and offer useful real-world evidence to supplement clinical trial data.

## Figures and Tables

**Figure 1:**
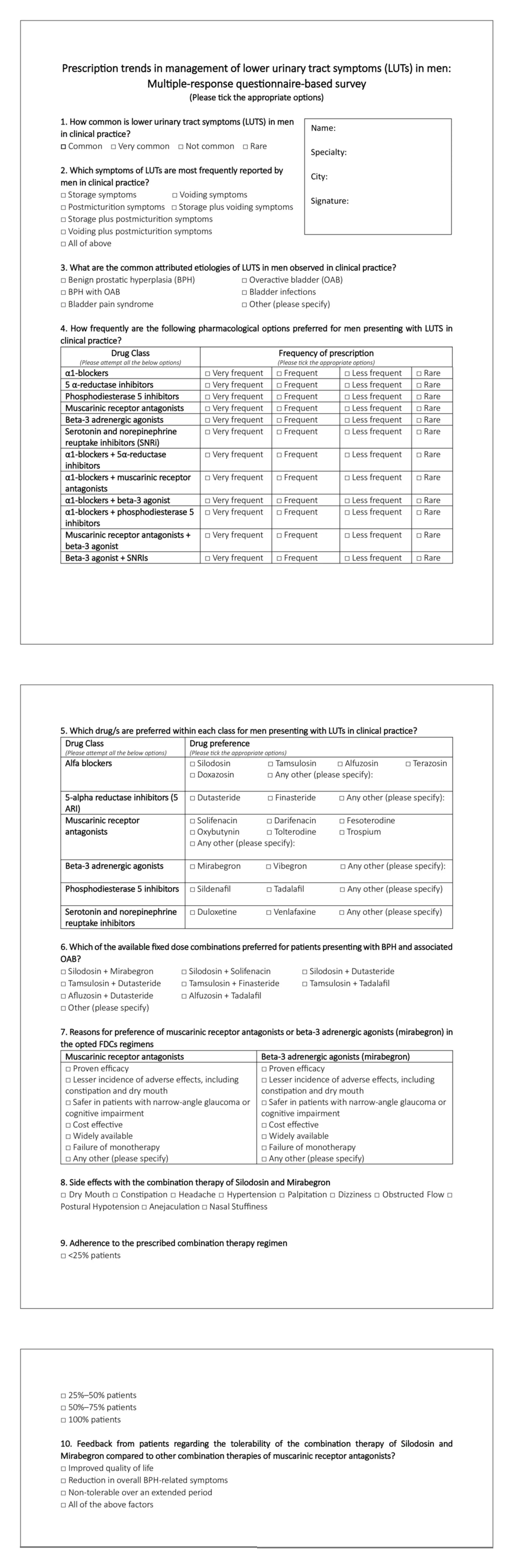
Study questionnaire
